# A Long Neglected Damper in the El Niño—Typhoon Relationship: a ‘Gaia-Like’ Process

**DOI:** 10.1038/srep11103

**Published:** 2015-07-21

**Authors:** Zhe-Wen Zheng, I.-I. Lin, Bin Wang, Hsiao-Ching Huang, Chi-Hong Chen

**Affiliations:** 1 Institute of Marine Environmental Science and Technology, National Taiwan Normal University, Taipei, Taiwan; 2 Department of Atmospheric Sciences, National Taiwan University, Taipei, Taiwan; 3 Department of Atmospheric Sciences, University of Hawaii, Honolulu HI 96822, USA

## Abstract

Proposed in the early 1970’s, the Gaia hypothesis suggests that our planet earth has a self-regulating ability to maintain a stable condition for life. Tropical cyclone (TC) is one of the earth’s most hazardous disasters; it is intriguing to explore whether ‘Gaia-like’ processes may exist in nature to regulate TC activities. El Niño can shift the forming position of the Western Pacific typhoons away from land. This shift enables typhoons to travel longer distances over ocean and is known to be a positive process to promote TCs to achieve higher intensity. What is neglected, however, is that there co-exists a negative process. Here we show that during El Niño, typhoons intensify over region undergoing strong ocean subsurface shoaling where upper ocean heat content can drop by 20–50%. This ‘worsen’ ocean pre-condition can effectively reduce ocean’s energy supply for typhoon intensification during typhoon-ocean interaction. We find this an elegant, ‘Gaia-like’ process demonstrating nature’s self-regulating ability. Though during El Niño, typhoons can take advantage of the longer travelling distance over ocean to achieve higher intensity, nature is also providing a damper to partially cancel this positive impact. Without the damper, the situation could be even worse.

With 20–30 tropical cyclones (TCs) formed and intensified each year, Western North Pacific Ocean (WNPO) is the most energetic and hazardous TC basin in the world. Known as typhoons, each year these TCs impose threats to a billion population and mega volume (US $5 billion yr^−1^) of economical activities in Asia^1^,^2^. During El Niño years, typhoon activities (e.g. number, life time, track, rainfall, landfall position, and forming position) can be greatly altered and results in differences in consequential damage and impacts^3^,^4^,^5^,^6^,^7^,^8^,^9^,^10^,^11^,^12^,^13^,^14^,^15^,^16^,^17^,^18^. It is thus important to understand the El Niño-typhoon relationship; especially climate projections have suggested a possible El Niño-like future scenario for Pacific under global warming^16^. Though many aspects have been studied, there is one aspect which has long been neglected. That is the relationship between ocean’s subsurface thermal condition, El Niño, and typhoon intensification.

In the current research framework, it is generally understood that because El Niño causes large shift of TC’s forming (genesis) position to the southeast (Fig. 1), TCs can travel longer distance over ocean before encountering the Asia Pacific continents to achieve higher intensity^4^,^5^,^6^,^7^,^8^,^9^. For example, the averaged genesis position and the averaged life-time peak intensity of the 1997 El Niño year (composite of 8 recent El Niño events, see Methods) was ~171.01°E, 8.94°N (154.68°E, 12.5°N) and 53.3 (46.8) ms^−1^. In comparison, the long-term climatological genesis position and the peak intensity was 146.98°E, 14.55°N (i.e. closer to land), and 43 ms^−1^ (not as intense), respectively (details see supplementary).

However, for long the above framework neglects the fact that ocean subsurface condition is also different under El Niño. In El Niño years, strong shoaling takes place in the subsurface of the WNPO^3^. As a result, ocean thermocline is not as deep and upper ocean heat content reduces (Figs. 1, S1-S12). These are negative factors for TC intensification^19^,^20^,^21^,^22^,^23^,^24^. In other words, though indeed typhoons can travel longer distance over ocean during El Niño, they are not travelling over the same ocean but over a ‘worsen’ (less-favourable) ocean (Figs. 1, S1-S12).

As will be shown, we demonstrate that there is an additional process from subsurface ocean taken place in the El Niño-typhoon intensity relationship. In contrary to the commonly-known positive facilitator related to the longer travelling distance, the subsurface factor is a damper which acts as brake to restrain typhoons from over-intensifying. This is found not only in the strong 1997 El Niño year, but also in the other El Niño years.

Ocean is the energy source for TC intensification, thus is a very important environmental factor for TCs^19^,^20^,^23^,^25^,^26^,^27^,^28^. Certainly atmospheric environment (e.g. wind shear) is also important, and has already been much discussed^9^,^12^. In this research, we focus on ocean’s energy supply perspective for TC intensification. To satisfy ocean’s condition, both sea surface temperature (SST) and ocean’s subsurface condition (typically from SST down to 200 m) are critical^21^,^22^,^23^,^24^,^25^,^26^,^27^,^28^,^29^,^30^,^31^,^32^,^33^. The warmer the pre-TC SST, the higher the pre-TC upper ocean heat content (UOHC, integrated heat content from SST down to the 26 °C isotherm (D26)), the thicker the pre-TC subsurface warm ocean layer (typically characterized by D26), the more favourable it is for intensification^15^,^16^,^17^,^18^,^19^,^20^,^21^,^22^,^23^,^24^,^25^,^26^,^27^,^28^.

This is because these more favourable pre-TC ocean conditions will later limit the TC-induced ocean cooling effect (i.e. more prohibition of subsurface cold water to be mixed to the ocean surface by the TC wind), during TC-ocean interaction^19^,^20^,^21^,^22^,^23^,^24^. As the cooling effect is weakened, more sensible and latent heat fluxes from the ocean for TC intensification correspond^19^,^20^,^23^,^25^,^26^,^27^,^28^. Reversely, the colder the pre-TC SST, the lower the pre-TC UOHC, the shallower the pre-TC ocean subsurface warm layer, the stronger the TC-induced cooling effect during intensification, the less fluxes will be for intensification.

As in Fig. 1b, during the 1997 El Niño, WNPO TCs are formed and intensified over region of evident reduction in heat content of ~20–50 kJ cm^−2^. As compared to the normal heat content of >100 kJ cm^−2^ (Fig. S1), this is an evident drop by ~20–50%. Consistent results are found in other El Niño years. Fig. 1c illustrates the composite of 8 El Niño events since 1980, an average drop of ~10–30 kJ cm^−2^ (i.e., 10–30%) is found (details see Methods and supplementary).

Given the strong pre-existing subsurface shoaling over the western North Pacific Ocean during the El Niño TC season, we conducted numerical experiments to examine the possible consequential impact. Fig. 2a compares the pre-TC initial ocean vertical temperature profiles during the 1997 El Niño and the ‘if no shoaling’ scenario (based on ocean climatology from 1980–2010), along the same track. It can be seen that during El Niño, colder subsurface water was much closer to the ocean surface (green profile), as compared to the no-shoaling scenario (purple profile). Consistent results based on the composites of the 8 recent El Niño events are also found, and depicted in the supplementary (Fig. S13).

Using profiles in Fig. 2a as input to the 3D Price-Weller-Pinkel ocean mixed layer model^29^, we test how this difference in the initial ocean subsurface pre-condition could impact TC-ocean interaction and ocean’s energy supply for TC intensification (Methods). We first examine the simulated TC-induced ocean cooling effect under different categories of TC wind speeds. From Fig. 2b, one sees that under the same TC forcing (wind speed), cooling effect is stronger from the El Niño profile (green curve), as compared to the ‘if no-shoaling’ condition (purple curve).

This stronger cooling effect can lead to smaller energy supply [Sensible Heat Flux (SHF) and Latent Heat Flux (LHF)] from ocean for TC’s intensification^19^,^20^,^22^,^23^,^27^,^28^,^29^,^30^, as seen below:


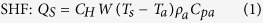



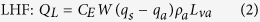


where *C*_*H*_ and *C*_*E*_ are the sensible and latent heat exchange coefficients^31^, *W* is TC wind speed, *T*_*S*_ and *T*_*a*_ are the during-intensification SST (i.e. pre-TC SST minus cooling effect, i.e. the temperature in Fig. 2b) and near surface air temperature, q_s_ and q_a_ are ocean surface and air specific humidity, *ρ*_*a*_, *C*_*pa*_, and *L*_*va*_ are air density, heat capacity of the air, and latent heat of vaporization.

From the above equations, one sees stronger cooling effect corresponds to smaller *T*_*S*_ (because *T*_*S*_ is the pre-TC SST minus cooling effect, Fig. 2b). If *T*_*S*_ (SST) is small, it means that ocean is *less warm* during TC-ocean interaction. The less-warm ocean thus supply less sensible heat flux (SHF) from ocean to TC because SHF is a function of the difference between atmosphere and ocean, i.e. *T*_*S*_-*T*_*a*_ (eqn. 1). Similar situation is also found for the latent heat flux (LHF) because LHF is a function of the air-sea humidity difference, (q_s_-q_a_). As *q*_*s*_ is a function of the during-TC SST (i.e. *T*_S_), smaller LHF and (q_s_-q_a_) corresponds. This reduction in the flux supply is found not only in the 1997 El Niño, consistent results are also found in the composites of other El Niño events (Figs. S13-S15).

The above results suggest that during El Niño, the shoaling effect creates a different initial ocean setting for TCs, thus can induce stronger cooling effect during TC’s intensification over ocean. Consequentially, there is less enthalpy (latent + sensible heat) flux to be supplied for intensification^19^,^20^,^22^,^23^. As in Table 1a, the estimated enthalpy flux supply based on the 1997 El Niño profile was about 30% less (in average 500 Wm^−2^) than the supply if there was no shoaling (~712 Wm^−2^), under the same atmospheric *T*_*a*_ and q_a_ input. This can be a substantial reduction in energy supply for TC intensification (i.e., a damper)^19^,^20^,^32^,^33^. If there was no pre-existing ocean subsurface shoaling, then given the long TC travelling distance (3066 km) during El Niño, the compounded distance-integrated flux supply would be 2.182 × 10^9^ Wm^−1^ (712 Wm^−2^ × 3066 km). This is a very large supply, equivalent to ~197% as compared to the normal supply of 1.127 × 10^9^ Wm^−1^ (normal supply is a reference base, from the long-term averaged flux and distance, see Fig. S16 and Table 1). However, because of the presence of the strong ocean subsurface shoaling during El Niño, the distance-integrated flux supply can be effectively reduced to 1.534 × 10^9^ Wm^−1^ (500 Wm^−2^ × 3066 km, Table 1a), equivalent to ~136% as compared to normal.

In other words, though during El Niño, the total distance-integrated flux supply is still higher than normal (Table 1a, 136%, consistent with the observed higher TC intensity during El Niño^4^,^5^,^6^,^7^,^8^,^9^,^10^), it is already a ‘discounted’ supply. If without the working of the subsurface damper, there could be even higher (197% of normal) energy supply, i.e. an even more severe scenario. This situation is found not only in the 1997 El Niño, but also in the other El Niño events. Results from the composite of the 8 El Niño events since 1980s (1982, 1986, 1987, 1991, 1994, 1997, 2002, 2004) show that the averaged total distance-integrated flux supply is 1.434 × 10^9^ Wm^−1^ (127% of normal). However, if there was no subsurface damper, the supply would be even higher, i.e. going up to 1.676 × 10^9^ Wm^−1^ (149% of normal) (Table 1b and supplementary).

This research shows that nature is more complex than originally perceived in the current research framework. It appears to be similar to the Gaia hypothesis which states nature’s self-regulating ability^34^,^35^. As illustrated in Fig. 3, for long the linkage between El Niño and typhoon’s intensification focuses on the positive impact due to the genesis position shift and longer travelling distance (red linkages)^4^,^5^,^6^,^7^,^8^,^9^,^10^. In this research, we show that the linkage is more complex and there is a co-existing negative linkage from subsurface shoaling (blue linkages). This negative linkage can effectively offset part of the positive impact and can be viewed as a protective measure from nature to restrain TCs from intensifying without constraint.

Otherwise, if the ocean was to remain the same, then given the much-longer travelling distance during El Niño, the total distance-integrated flux supply would be extremely high (for example in the 1997 El Niño, reaching 197% (nearly double) as the normal supply). However, because nature provides a ‘worsen’ ocean along TC’s intensification track, a co-existing negative (damping) effect can be effectively at work to cancel a large (though not the entire) part of the positive effect. As a result, during El Niño, typhoons could still be more intense than normal, since the residual distance-integrated flux supply after offset is still higher than normal (136% of normal, Table 1a). However, without such partial cancellation effect, the situation could likely be even more severe. Though no biosphere is involved, it appears to be a ‘Gaia-like’ self-regulating process through large-scale physical interaction in the earth’s ocean and atmospheric system^34^,^35^.

In recent years it has been suggested that there could be two different types of El Niño, i.e., the classical, Eastern-Pacific (EP) El Niño or the ‘Modoki’, Central-Pacific (CP) El Niño^11^,^12^,^13^,^14^,^17^. The result in Fig. 1c is based on the composites of both types of El Niño events. If examining them separately, consistent results are also found, though the magnitude of subsurface shoaling is stronger in the EP than in the CP events (Figs. S3, S4, S14, S15).

There are interesting issues to be explored in the future research. For instance, whether other climate modes (e.g. the Indian Ocean Dipole (IOD)) may further modify the observed process would be interesting. Though the 1997 event was one of the strongest El Niño ever observed, IOD was also active in that year^13^. It has been suggested that through modification of the atmospheric processes, IOD in 1997 could modulate the TC activity over the western North Pacific^13^. It would be interesting to explore whether IOD could also contribute to the ocean shoaling process and subsequently impact TCs. Finally, climate projections have suggested an El Niño-like future scenario for Pacific under global warming^16^. How this ocean subsurface damper will evolve will also be an interesting research topic to pursue.

## Methods

### Data sets

#### a. Ocean surface and subsurface temperature data

Ocean Reanalysis System 4 (ORAS4) data of the European Centre for Medium-Range Weather Forecasts (ECMWF) was used. This is a monthly, one-degree grid reanalysis ocean data set. Sea Surface Temperature (SST), depth of the 26 °C isotherm (D26), Upper Ocean Heat Content (UOHC, integrated heat content from SST down to D26), and ocean depth-temperature profiles were all obtained or derived from this data set.

#### b. Sea Surface Height Anomaly (SSHA) data

Monthly, delayed-mode, gridded, multi-altimetry SSHA data from the AVISO (Archiving, Validation and Interpretation of Satellite Oceanographic Data) data base (http://www.aviso.oceanobs.com/) was used.

#### c. Near surface atmospheric temperature (*T*_*a*_) and humidity (*q*_*a*_) data

The atmospheric data was based on the monthly data from the ECMWF (European Centre for Medium-Range Weather Forecasts)’s Interim Reanalysis database at each 1.5 degree grid.

#### d. TC track and intensity data

TC track and intensity data was from the US Joint Typhoon Warning Center (JTWC)’s Best Track data base (obtained via the web site of Professor Kerry Emanuel, Massachusetts Institute of Technology, USA). TC intensity categories are defined from the Saffir–Simpson TC scale (in 1-min maximum sustained wind speed): category 1: 34–43 m s^−1^, category 2: 44–50 m s^−1^, category 3: 51–59 m s^−1^, category 4: 59–71 m s^−1^, category 5: > 71 m s^−1^. The long-term climatological mean genesis and intensity peak (i.e. life-time maximum of a TC) locations were calculated based on the average of all western North Pacific TCs (746 cases), from August to November of each year, between 1958 and 2010. The mean intensification track was obtained through a 2-D polynomial regression from the individual intensification track locations of each TC (i.e., the track between the genesis and peak locations) of the 746 cases (details see supplementary). Similarly, the mean genesis location, mean peak location, and mean intensification track was obtained for the El Niño years.

### TC season

as El Niño peaks towards winter, the corresponding TC season in this study is defined as from August to November (ASON).

### El Niño events

8 El Niño events since 1980s (1982, 1986, 1987, 1991, 1994, 1997, 2002, 2004) are included in this study^11^,^12^,^13^,^14^,^17^. The EP El Niño events are 1982, 1987, and 1997. The CP El Niño events are 1986, 1991, 1994, 2002, 2004^11^,^12^,^13^,^14^,^17^. The reason to start from 1980 is due to the quality of the ocean subsurface reanalysis data. Subsurface ocean data is a key component in this research but prior to 80’s, there were very few *in situ* ocean subsurface observations to be included in the reanalysis data. Only from the 80’s, there were more subsurface measurements, e.g., from the TAO (Tropical Atmosphere Ocean) array (from mid 80’s) and Argo floats (2000).

### During-intensification SST cooling estimation

**Figure 2:**
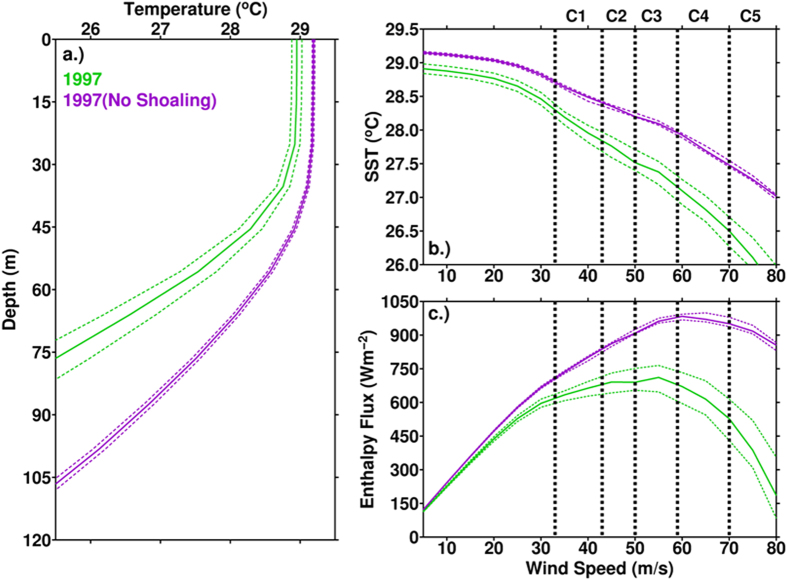
(**a**) The initial, pre-TC ocean depth-temperature profile (green profile) averaged along the TC mean intensification track (location see in Fig. 1b) in ASON 1997. For comparison, the purple profile is the initial profile under the ‘if no-shoaling’ scenario (based on the 1980–2010 ASON average), obtained along the same track. The dashed-profiles are the corresponding 95% confidence interval profiles from the mean. (**b**) During-intensification (from category-1 to 5 in the Saffir-Simpson scale) SST cooling as estimated using the Price-Weller-Pinkel 3-D ocean mixed layer model^29^. The initial input was based on the 2 profiles in (**a**). (**c**) As in (**b**), but for the corresponding air-sea enthalpy (latent + sensible) flux supply. The results based on the composites of El Niño events are in Fig. S13-S15.

During-intensification SST cooling (Figs. 2b, S13b-S16b) was estimated using the 3D Price-Weller-Pinkel ocean mixed-layer model^29^. This is a well-known model for the TC-induced ocean response simulation^23^,^24^. It solves for the wind-driven, baroclinic ocean response, including a treatment of turbulent vertical mixing in the upper ocean. The horizontal resolution was 5 km and the vertical resolution was 5 m. Simulations were performed from category-1 to 5 in the Saffir-Simpson TC Scale. Based on the different initial pre-TC ocean thermal profiles (e.g. Fig. 2a), the corresponding ocean-cooling effect under different wind speed was simulated. As ocean cooling effect is also a function of the TC translation speed^21^,^30^, the simulation was performed based on the long-term climatological mean translation speed (5 m s^−1^) of the western North Pacific TCs^28^.

### Air-sea flux calculation

as in the main text, SHF and LHF are calculated separately from the bulk aerodynamic formula. The SST input was from the output of the 3DPWP simulation, i.e. the SST under the corresponding TC wind (e.g. Fig. 2b) during TC-ocean interaction. *T*_*a*_ and *q*_*a*_ are from the ECMWF data (point 1c). The exchange coefficients were from the CBLAST field experiment^31^. As in Fig. 2c (and Figs. S13-15), given the same TC translation speed and wind speed conditions, the shallower the initial (pre-TC) thermal profile (i.e., under the El Niño condition), the stronger the cooling effect, the smaller the sensible and latent heat fluxes correspond.

### Statistical analyses

Statistical significance test was performed based on the two-sample T-test. Uncertainty in the mean is assessed based on the 95% confidence interval (CI). Details and results see supplementary.

## Additional Information

**How to cite this article**: Zheng, Z.-W. *et al.* A Long Neglected Damper in the El Niño —Typhoon Relationship: a ‘Gaia-Like’ Process. *Sci. Rep.* **5**, 11103; doi: 10.1038/srep11103 (2015).

## Supplementary Material

Supplementary Information

## Figures and Tables

**Figure 1:**
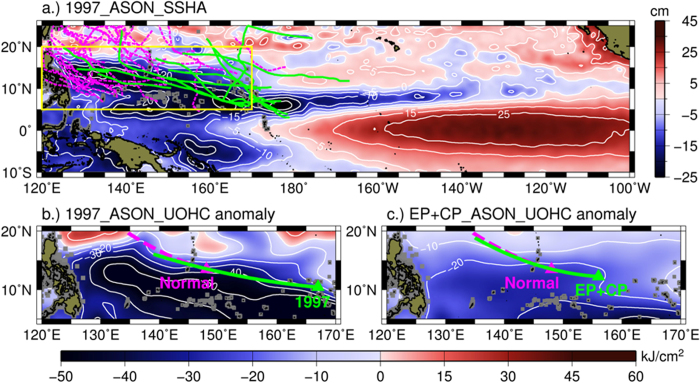
(**a**) During-El Niño Sea Surface Height Anomaly (SSHA) map of the tropical Pacific Ocean, as observed by satellite altimetry in August-November (ASON) 1997. The corresponding TC intensification tracks [from the genesis position to the life-time intensity peak] are depicted in green. For comparison, the non-El Niño TC intensification tracks (ASON of 1998–2001) are depicted in pink. The south-eastward shift of TC tracks during El Niño is evident. The study region is shown by yellow box. (**b**) Upper ocean heat content (UOHC) anomaly [with respect to the 1980–2009 mean] of the study region in the 1997 TC season (ASON). Data source: ECMWF ORAS4 reanalysis data. The corresponding mean TC intensification track and the genesis position (in triangle) are illustrated in green. For comparison, the normal (long-term) TC intensification mean track and genesis position (triangle) are depicted in pink. (**c**) As in (**b**), but for the UOHC anomaly based on composites of 8 El Niño events. Figures are generated using the GMT (https://www.soest.hawaii.edu/gmt/) and IDL softwares (http://www.exelisvis.com/ProductsServices/IDL.aspx).

**Figure 3:**
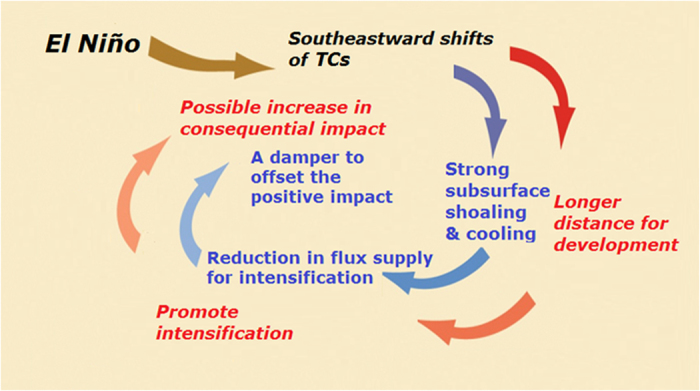
A schematic diagram illustrating the Gaia-like mechanism^34^,^35^ in the El Niño—TC (typhoon) intensity relationship. The linkages in red are for the positive impact and the linkages in blue are for the negative impact from the subsurface damper.

**Table 1:** Summary of ocean’s energy supply for TC intensification and the intensification distance: the during-intensification averaged enthalpy flux (SHF + LHF) is in the 2^nd^ column.

(a)	Flux (Wm^−2^)	Distance (km)	Flux × Distance (10^9^ Wm^−1^)	Flux × Distance .wrt. normal (%)	Genesis Position
***1997 El Niño***	*500 (46)*	*3066*	*1.534 (0.142)*	*136%*	*171.01*°*E 8.94*°*N*
***1997 El Niño (if no shoaling)***	*712 (10)*	*3066*	*2.182 (0.029)*	*194%*	*171.01* °*E 8.94* °*N*
					
**(b)**	***Flux (Wm***^***−2***^)	***Distance (km)***	***Flux × Distance (10***^***9***^***Wm***^***−1***^)	***Flux × Distance .wrt. normal (%)***	***Genesis Position***
***8 El Niño composites***	*597 (16)*	*2401*	*1.434 (0.039)*	*127%*	*154.68* °*E 12.5* °*N*
***8 El Niño composites (if no shoaling)***	*698 (7)*	*2401*	*1.676 (0.017)*	*149%*	*154.68* °*E 12.5* °*N*
					
**(c)**	***Flux (Wm***^***−2***^)	***Distance (km)***	***Flux*** ***×*** ***Distance (10***^***9***^***Wm***^***−1***^)	***Flux*** ***×*** ***Distance .wrt. normal (%)***	***Genesis Position***
***Normal condition***	*583 (16)*	*1932*	*1.127 (0.031)*	*100%*	*146.98* °*E 14.55* °*N*

The uncertainty estimates are in brackets, calculated based on the upper and the lower-bounded (95% confidence interval from mean) profiles in Fig. 2a. The averaged intensification distance is in the 3^rd^ column. The distance-multiplied flux supply is in the 4^th^ column and the percentage of the distance-multiplied flux supply with respect to (.wrt.) the normal condition is in the 5^th^ column.
