# Supplementary material for: A Long Neglected Damper in the El Niño—Typhoon Relationship: a ‘Gaia-Like’ Process
Source: Sci Rep. 2015 Jul 21;5:11103. doi: 10.1038/srep11103 (PMC4508847; doi:10.1038/srep11103)
Supplement: Supplementary Information [file srep11103-s1.doc]

Supporting Online Material for:

A Long Neglected Damper in the El Niño – Typhoon

Relationship: a ‘Gaia-Like’ Process

Zhe-Wen Zheng^1^, I.-I. Lin^2^*, Bin Wang^3^, Hsiao-Ching Huang^2^

,Chi-Hong Chen^2^

*^1^Institute of Marine Environmental Science and Technology,*

*National Taiwan Normal University, Taipei, Taiwan*

*^2^Department of Atmospheric Sciences, National Taiwan University, Taipei, Taiwan*

*^3^Department of Atmospheric Sciences, University of Hawaii, Honolulu HI 96822, USA*

**Correspondence to:* [***iilin@as.ntu.edu.tw***](mailto:iilin@as.ntu.edu.tw)

**1. UOHC, D26, and SST maps under different types of the El Niño conditions (Figs. S1-S12)**

Similar to Figs. 1bc in the main text, but for the complete series of UOHC, D26, and SST maps under different types of the El Niño conditions. The reference climatology (normal) condition is also depicted for comparison.

**2. Initial pre-TC profile, ocean cooling and flux estimation under normal and different types of the El Niño conditions (Figs. S13-S16)**

Similar to Fig. 2 in the main text, but for the results under the normal (Fig. S16) and different types of the El Niño conditions (Figs. S13-S15).

**3. Satellite altimetry sea surface height anomaly (SSHA) observation in 1997**

Figs. S17 and S18 illustrate the satellite SSHA observation in summer 1997. As in Fig. S17, the negative SSHA over the WNPO was already observed in July 1997, prior to the occurrence of the first supertyphoon Rosie of 1997. In other words, the negative SSHA pre-existed before Rosie’s passing and was not induced by typhoon. Fig. S18 shows the time-evolution of the development of this large-scale shoaling effect over the western Pacific in 1997, with negative SSHA strengthening from June to October. In contrary, over the eastern Pacific, positive SSHA developed in tandem.

**4. Statistical significance test of the ocean shoaling effect under different El Niño conditions**

Tables_supplementary_1-5 summarise the statistical results, comparing the ocean shoaling effect under different types of the El Niño conditions, with respect to climatology/normal. The null hypothesis is on the similarity between El Niño and normal (control). Confidence level is assessed using the calculated T values to examine the probability of rejecting the null hypothesis. It can be seen that for all types of the El Niño conditions, the cooling/shoaling situation (SST, D26 and UOHC) is also statistically significantly different from climatology, mostly ≥ 99.9%.

**5. Details of the mean track determination using the 2D polynomial regression method**

As in the Methods, 2-dimensional polynomial regression was used for the mean track retrieval. A number of quality control procedures were applied. They are (1) length check, (2) mean squared error check, so as to filter out highly irregular tracks, (3) composite mean track consistency check: all regressed coefficients for the mean track retrieval will be applied after filtering outliers (i.e. larger than or less than two standard deviation relative to their mean values).

**6. Further explanation on the TC-ocean interaction process, including UOHC, cooling effect and air-sea fluxes**

In the context of TC-ocean interaction and in this research, the upper ocean heat content (UOHC) is usually used to quantify the amount of the *pre-TC*, initial ocean heat ‘reservoir’ (Leipper and Volgenau 1972; Emanuel 1999; Bender and Ginis 2000; Shay et al. 2000; Cione et al. 2003; Lin et al. 2014). It is the energy ‘*available*’ for TC intensification, prior to TC’s passing. It is not the actually energy ‘*extracted* (utilised)’ by a TC during intensification. The actually energy extracted by a TC from ocean for is via the air-sea latent and sensible heat fluxes. This takes place ‘*during*’ TC-ocean interaction. During TC-ocean interaction and at the air-sea interface, sea surface temperature (SST) is cooled due to the vertical mixing process. This is because the intense TC wind mixes the deep, colder subsurface water to the air-sea interface and cools the SST. Because this cooling process cools the air-sea interface SST, it can subsequently affect the air-sea temperature and humidity difference, thus lead to impact on the flux supply.

Therefore, in the context of TC-ocean interaction and in this research, UOHC, cooling from TC-induced vertical mixing, and air-sea fluxes all need to be considered in a holistic manner. They are not separated entities but are related parameters, describing different components of the TC-ocean interaction process. The warmer the pre-TC SST, the higher the pre-TC UOHC, the less the during-TC induced ocean cooling effect, the more the sensible and latent heat flux from the ocean is available for TC intensification (Price 1981; Emanuel 1999; Bender and Ginis 2000; Shay et al. 2000; Cione et al. 2003; Lin et al. 2014; D’Asaro et al. 2014).

**7. Further statistical information for the El Niño TC cases**

As in the Methods, the TC season here is defined as ASON. The sample numbers of the 1997 El Niño (composite of the 8 recent El Niño events) are 14 (118). The corresponding mean and standard deviation of the TC genesis position, as well as the peak intensity were 171.01 ± 17.51 ∘E, 8.94 ± 4.88∘N (154.68 ± 17.43 ∘E, 12.50∘± 5.4 ∘N), and 53.3 ± 26.9 ms^-1^ (46.8 ± 20.3 ms^-1^). The long-term climatological genesis position was 146.98 ± 15.36 ∘E, 14.55 ± 5.78∘N (based on 746 TCs from ASON of 1958-2010). The long-term climatological peak intensity was 43.0 ± 19.9 ms^-1^. Using the statistical two-sample T test, the confidence level testing the difference between the mean TC peak intensity of the 1997 El Niño (composite of the 8 recent El Niño events ) from the long-term climatology is 84% (94%).

**Supplementary Figures:**


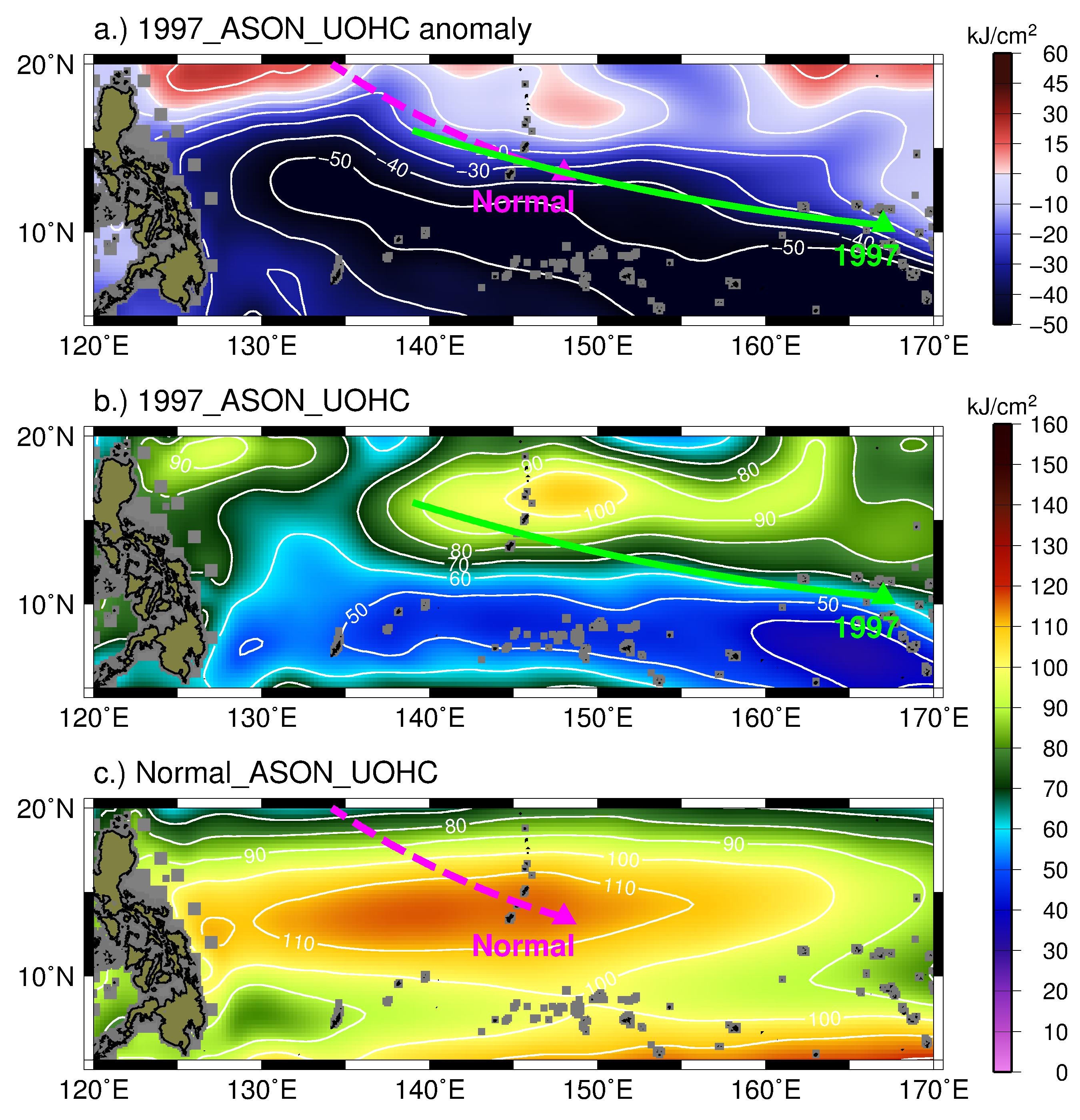
Fig. S1. **(a)** Upper ocean heat content (UOHC) anomaly [with respect to the 1980-2009 mean] of the study region in the 1997 TC season (ASON). Data source: ECMWF ORAS4 reanalysis data. The corresponding mean TC intensification track and the genesis position (in triangle) are illustrated in green. For comparison, the normal (long-term) TC intensification mean track and genesis position (triangle) are depicted in pink. **(b)** As in (a), but for the UOHC in the 1997 ASON. (c) As in (b), but for the UOHC from the average of 1980-2009, indicative of the normal condition.


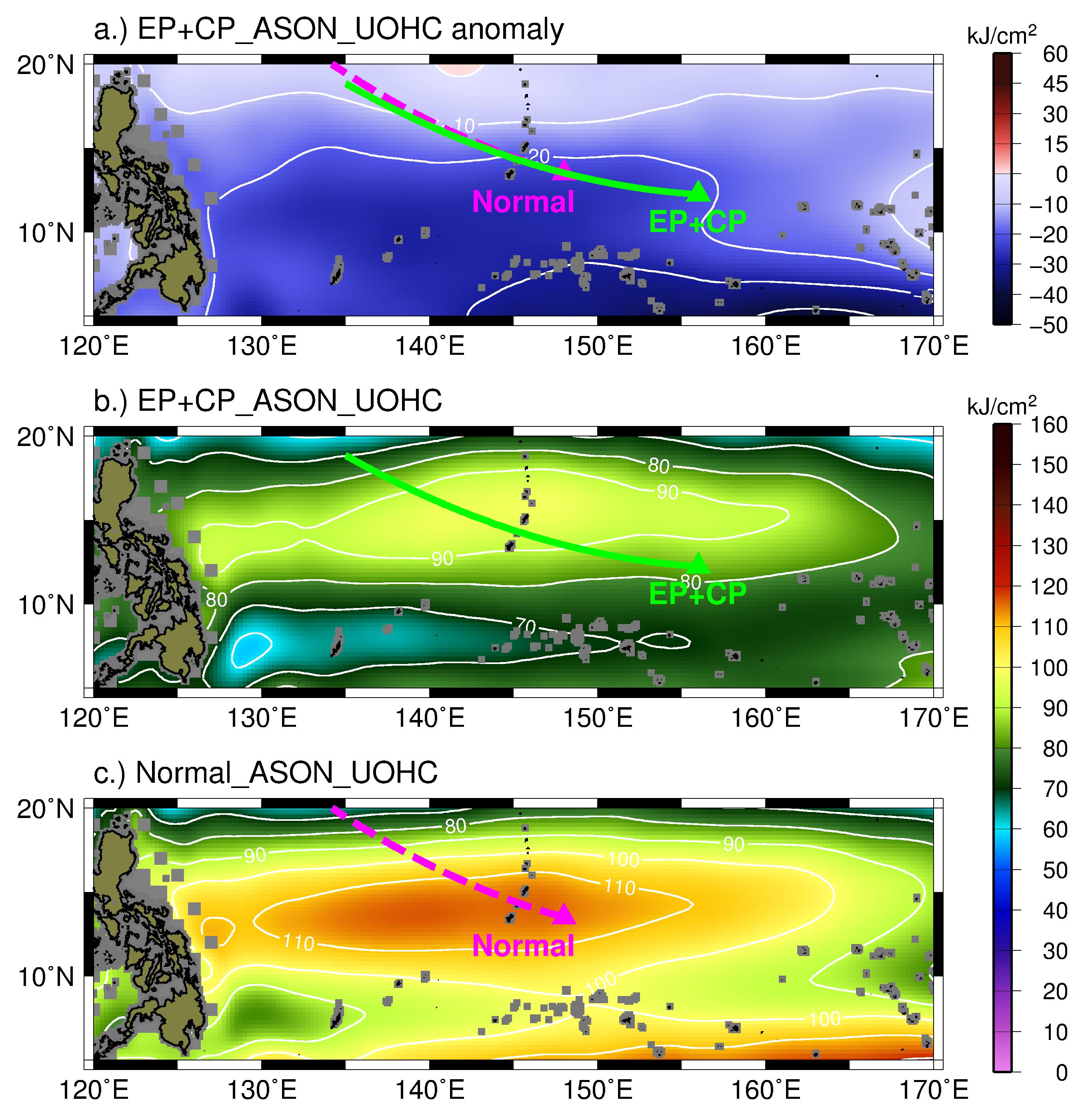
Fig. S2. As in Fig. S1, but for the composites of the 8 El Niño events (1982, 1986, 1987, 1991, 1994, 1997, 2002, 2004).

**
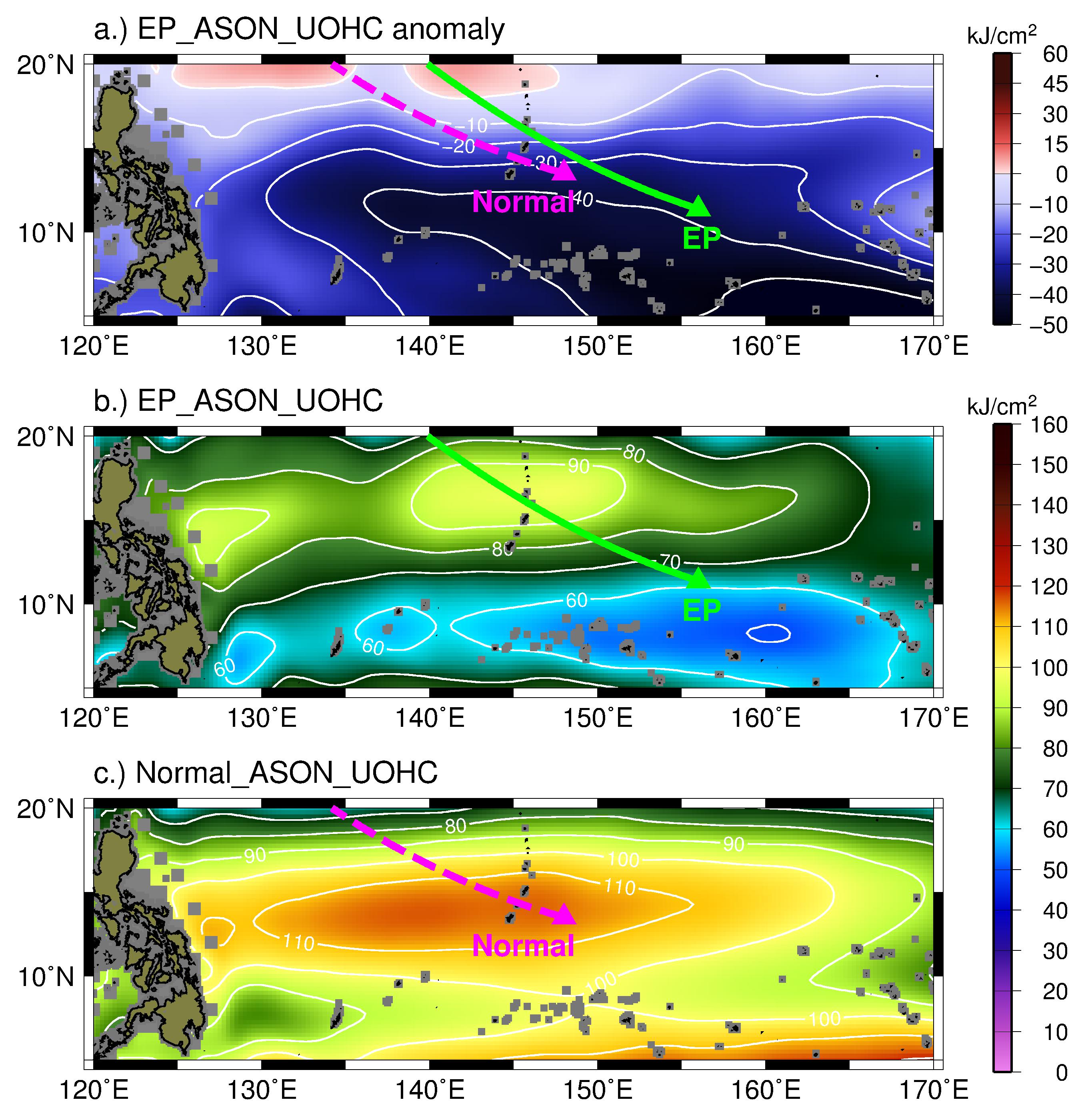
**

Fig. S3. As in Fig. S1, but for the composites of the 3 EP El Niño events (1982, 1987, 1997).


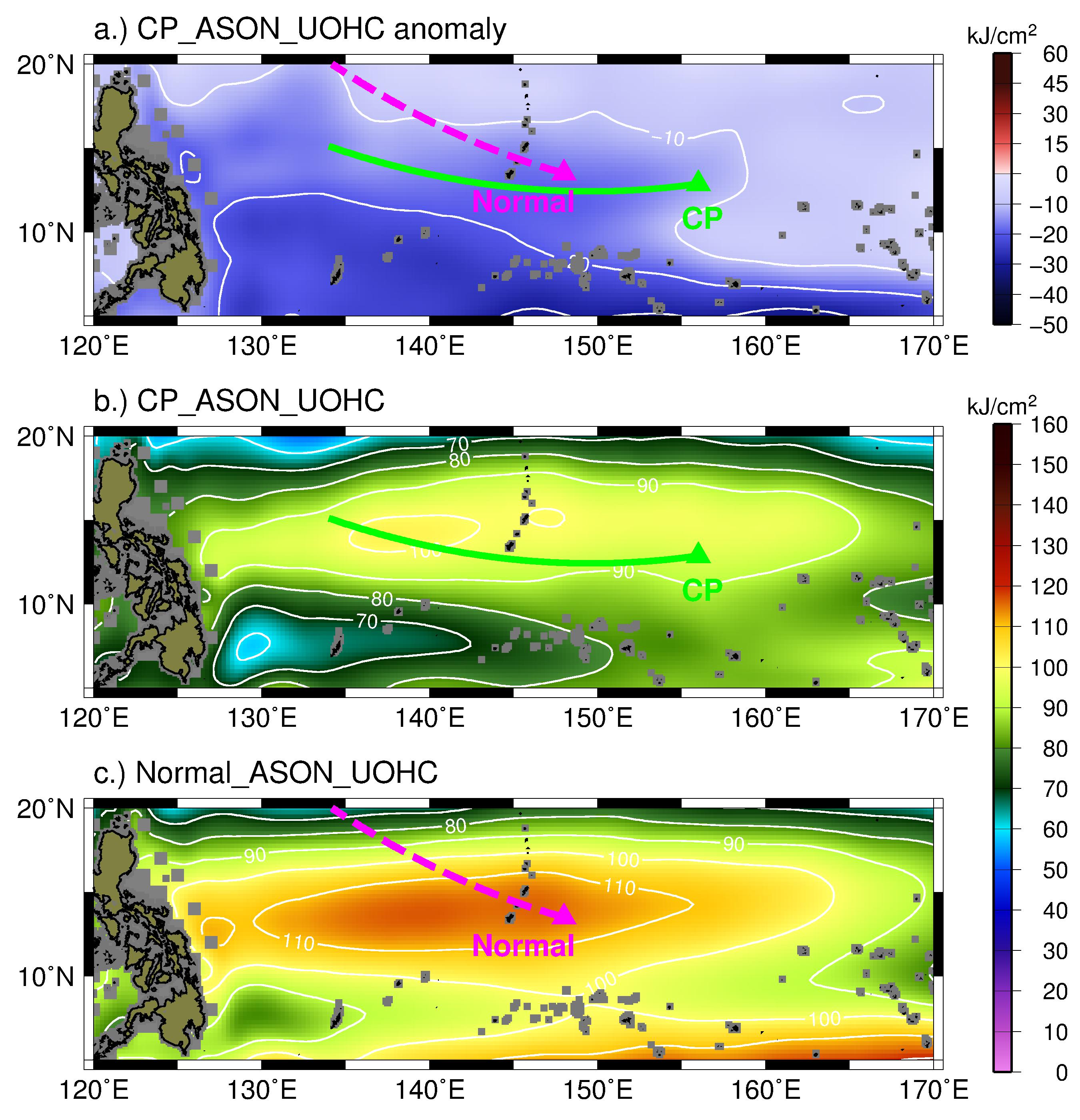
Fig. S4. As in Fig. S1, but for the composites of the 5 CP El Niño events (1986, 1991, 1994, 2002, 2004).


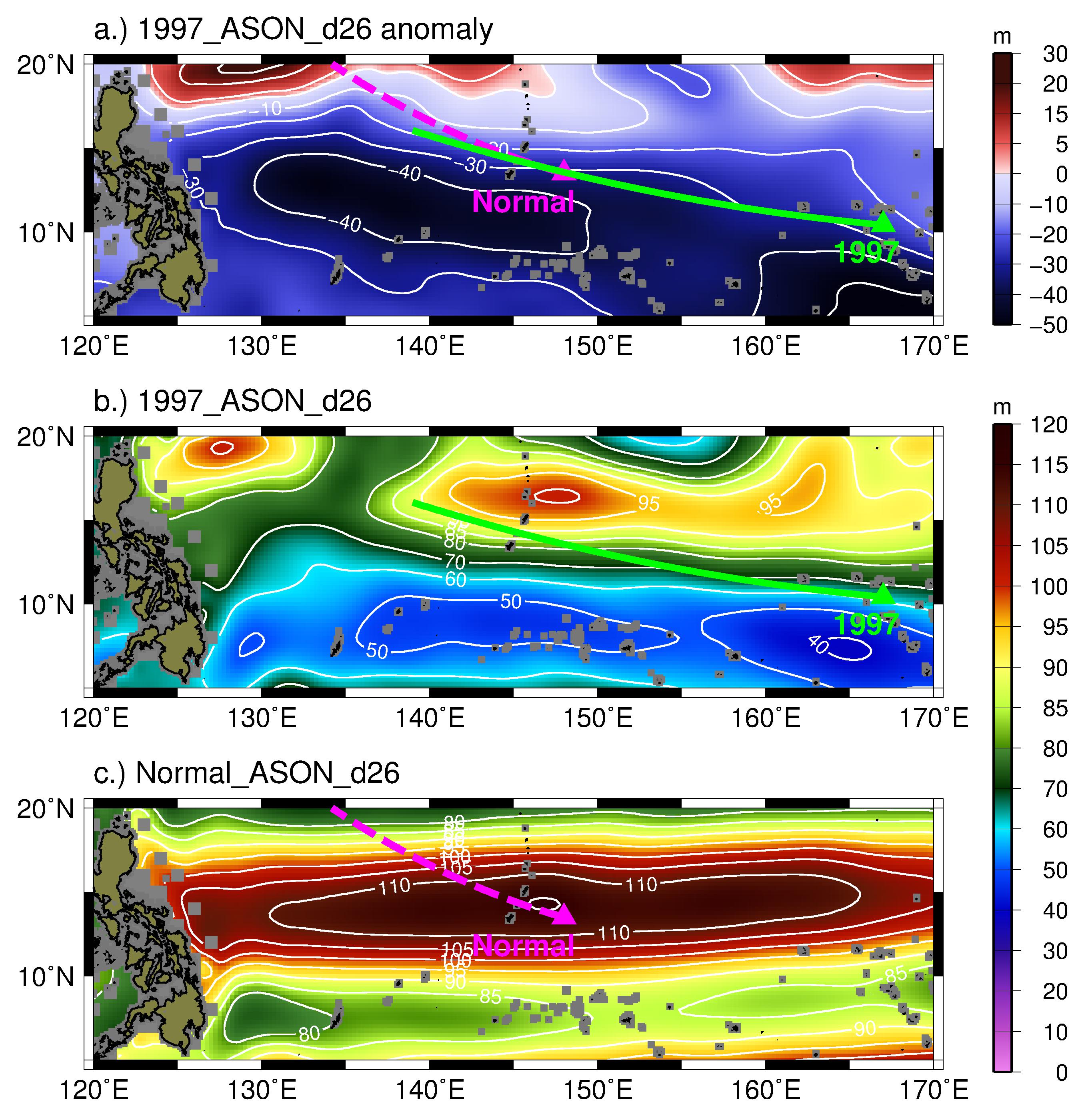
Fig. S5. As in Fig. S1, but for the 1997 D26.


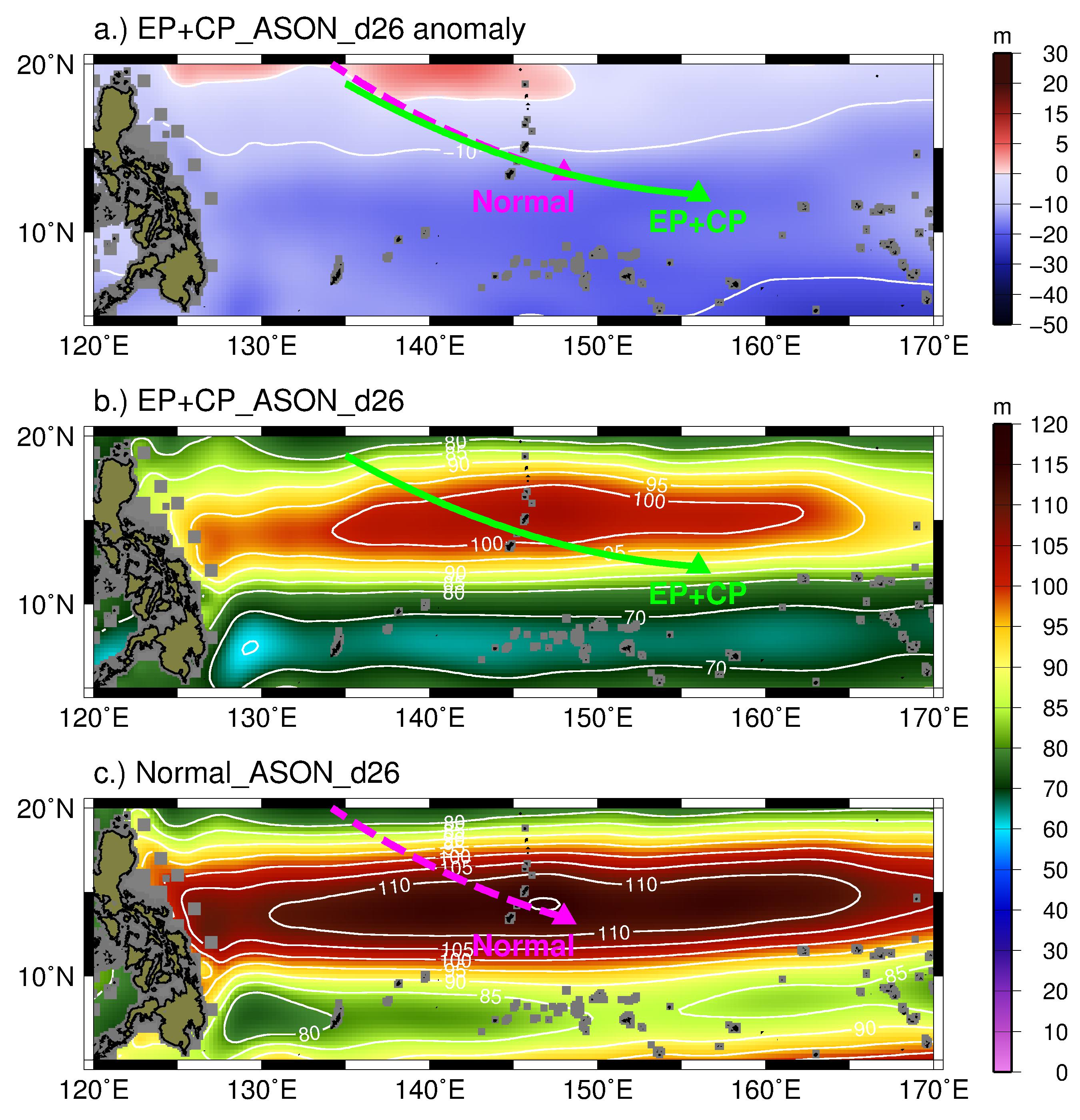
Fig. S6. As in Fig. S2, but for the D26 of the 8 El Niño composites.


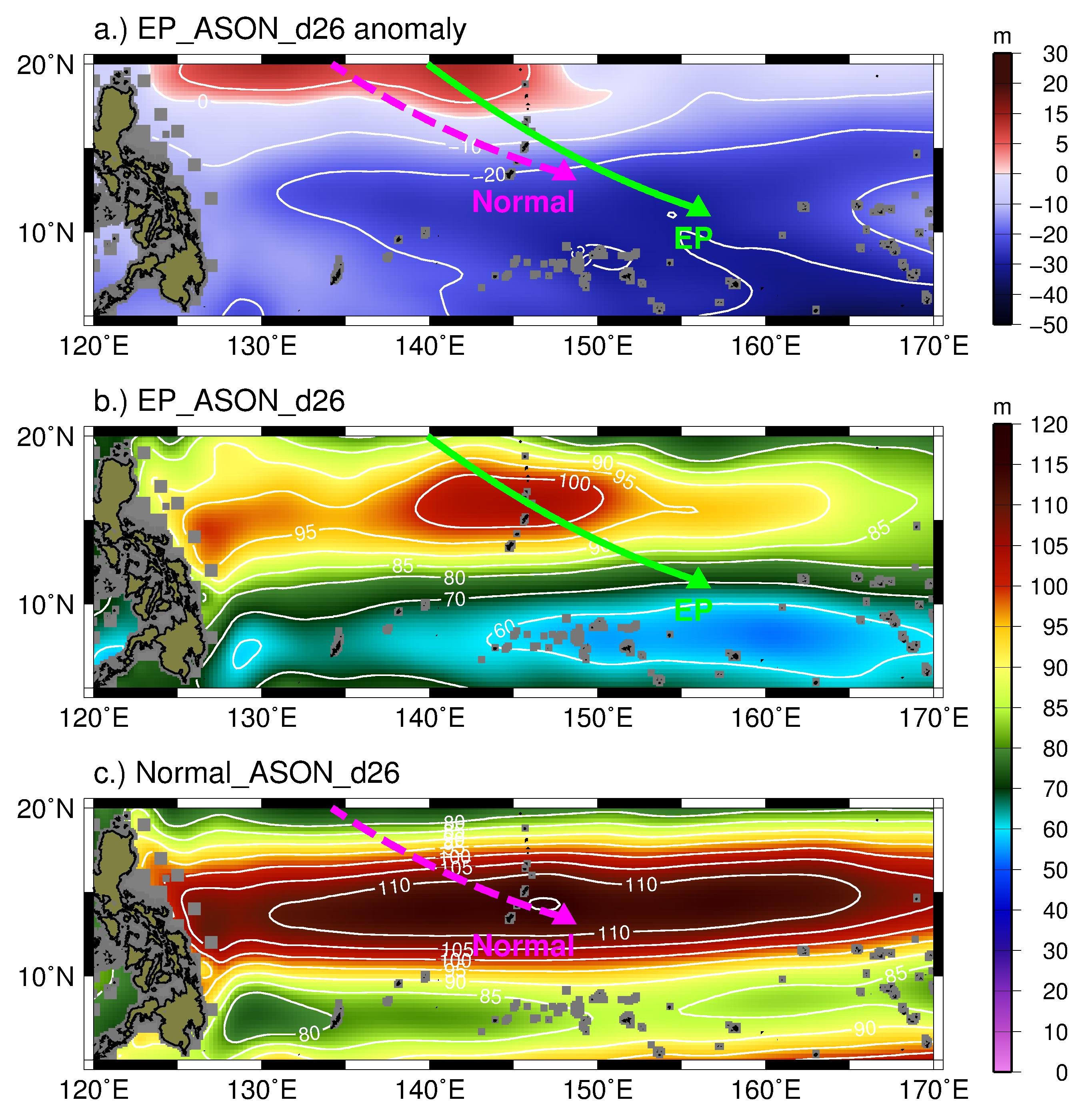
Fig. S7. As in Fig. S3, but for the D26 of the 3 EP El Niño composites.


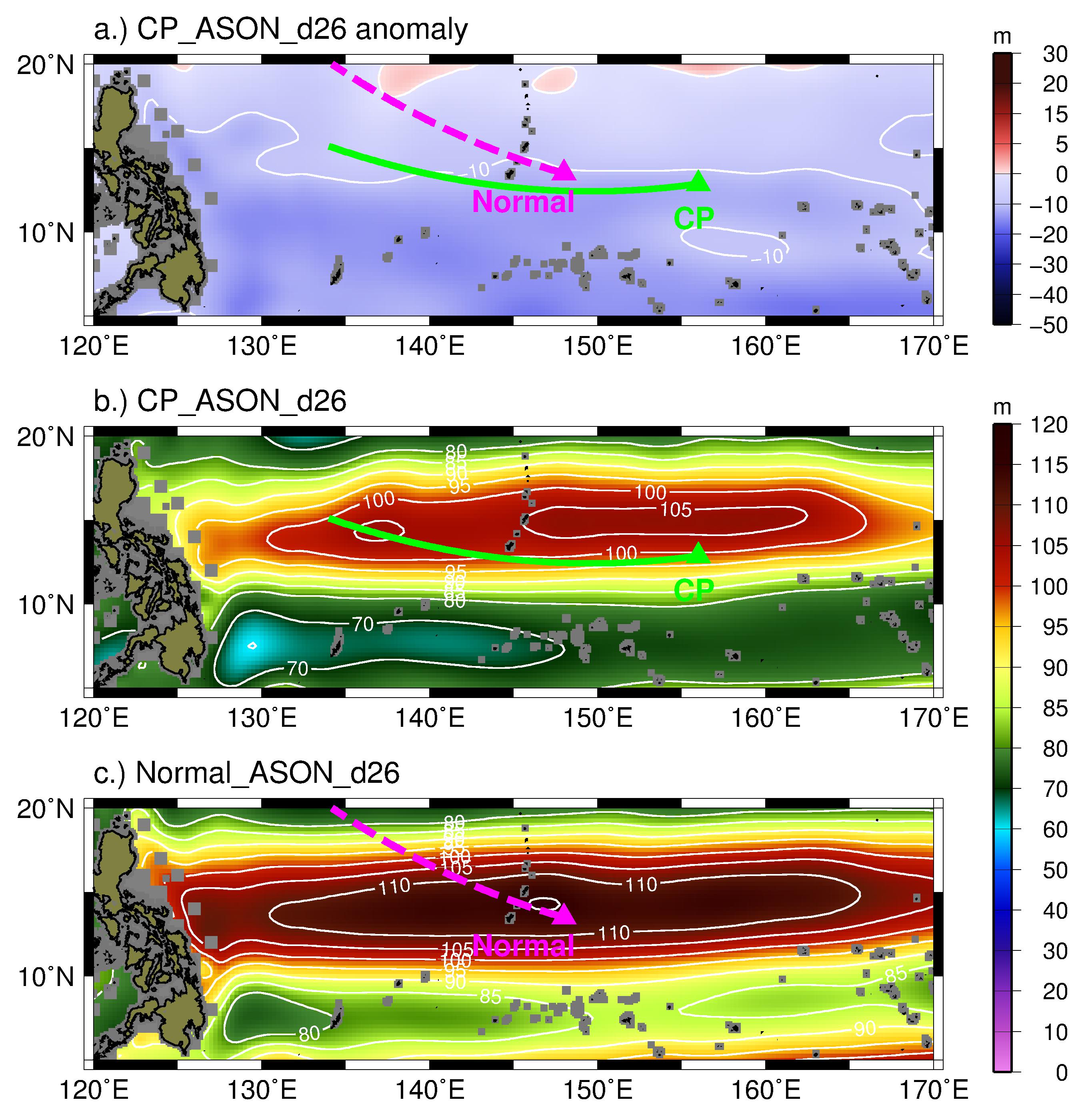
Fig. S8. As in Fig. S4, but for the D26 of the 5 CP El Niño composites.


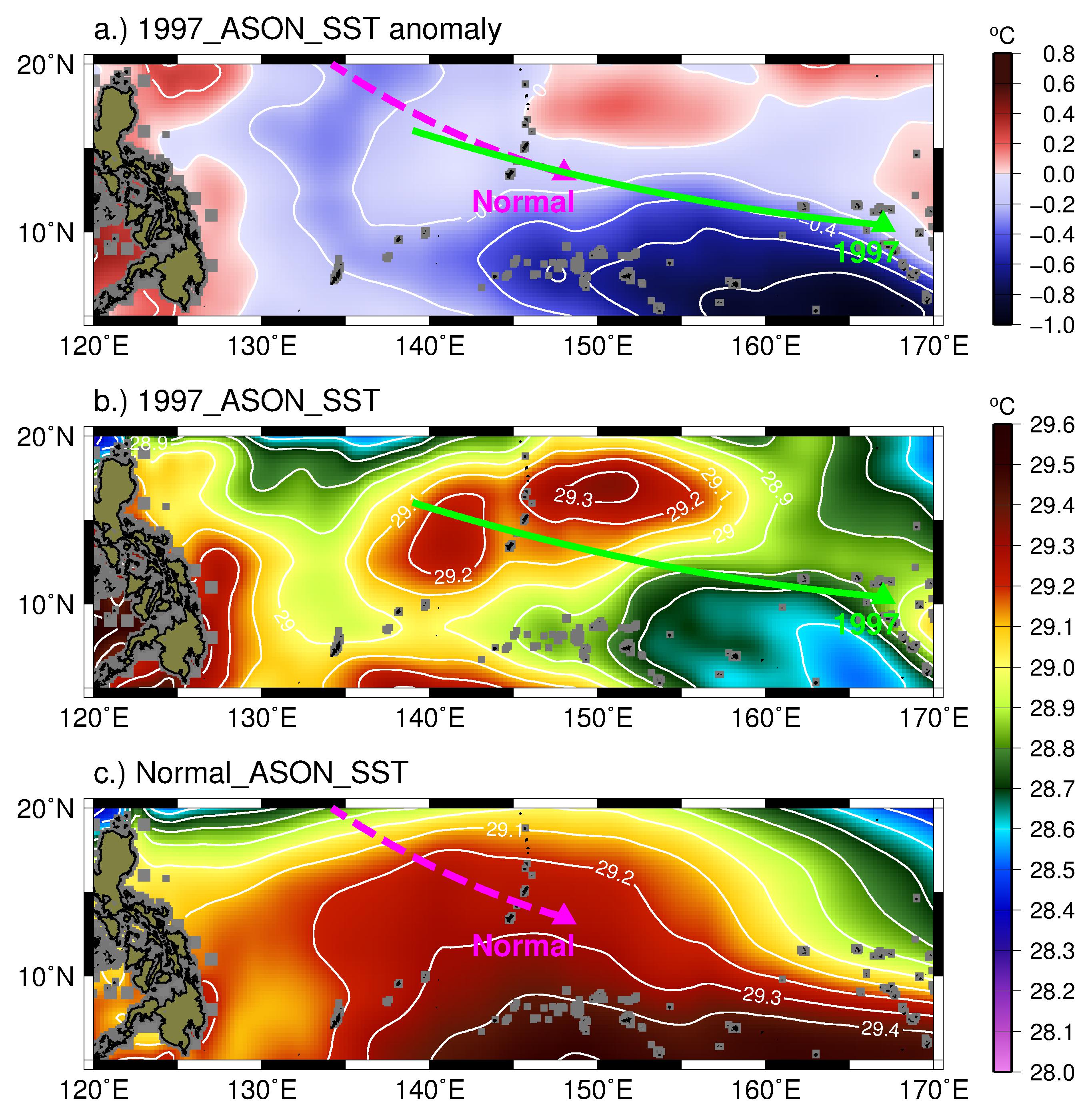
Fig. S9. As in Fig. S1, but for the 1997 SST.


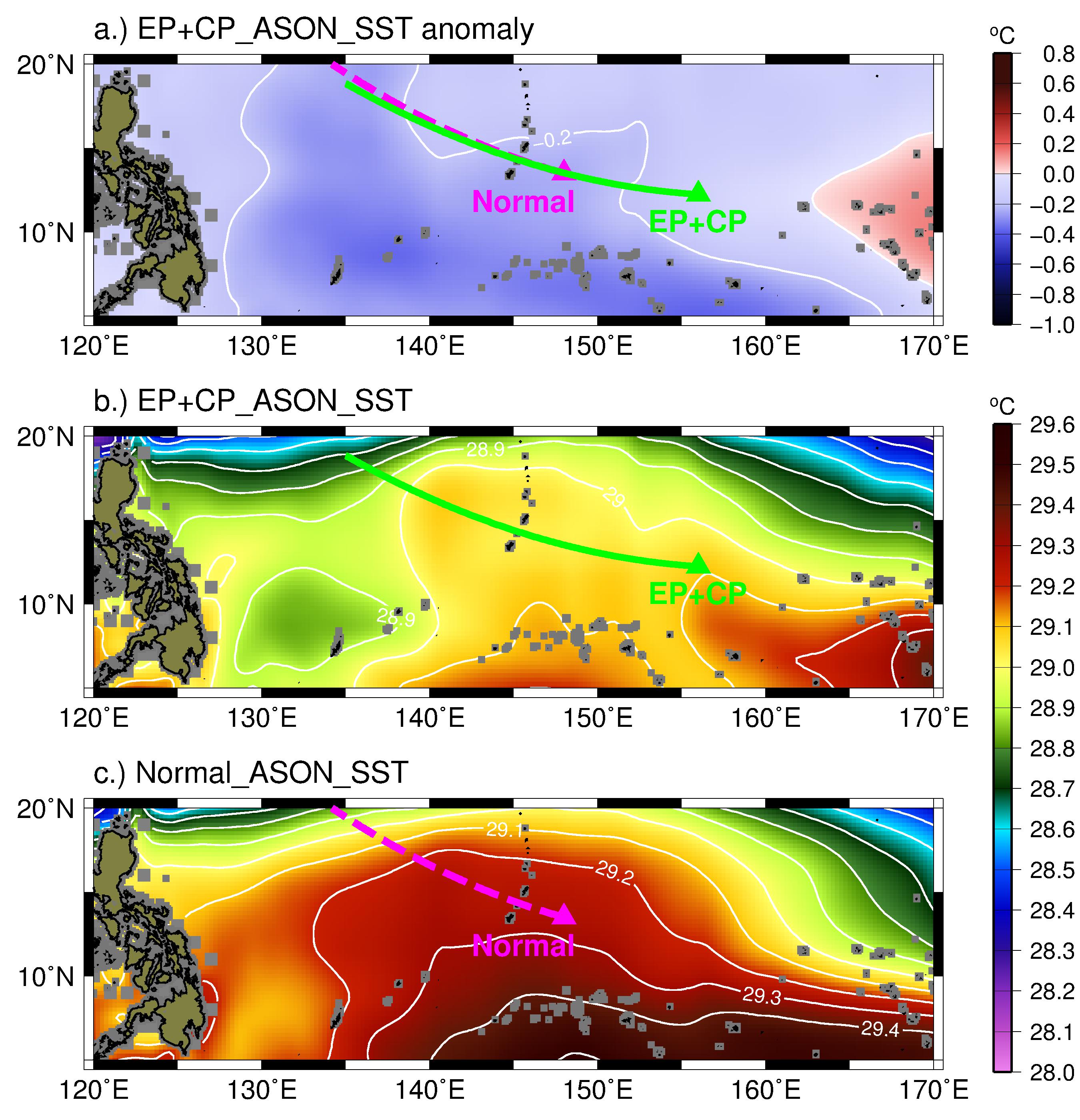
Fig. S10. As in Fig. S2, but for the SST of the 8 El Niño composites.


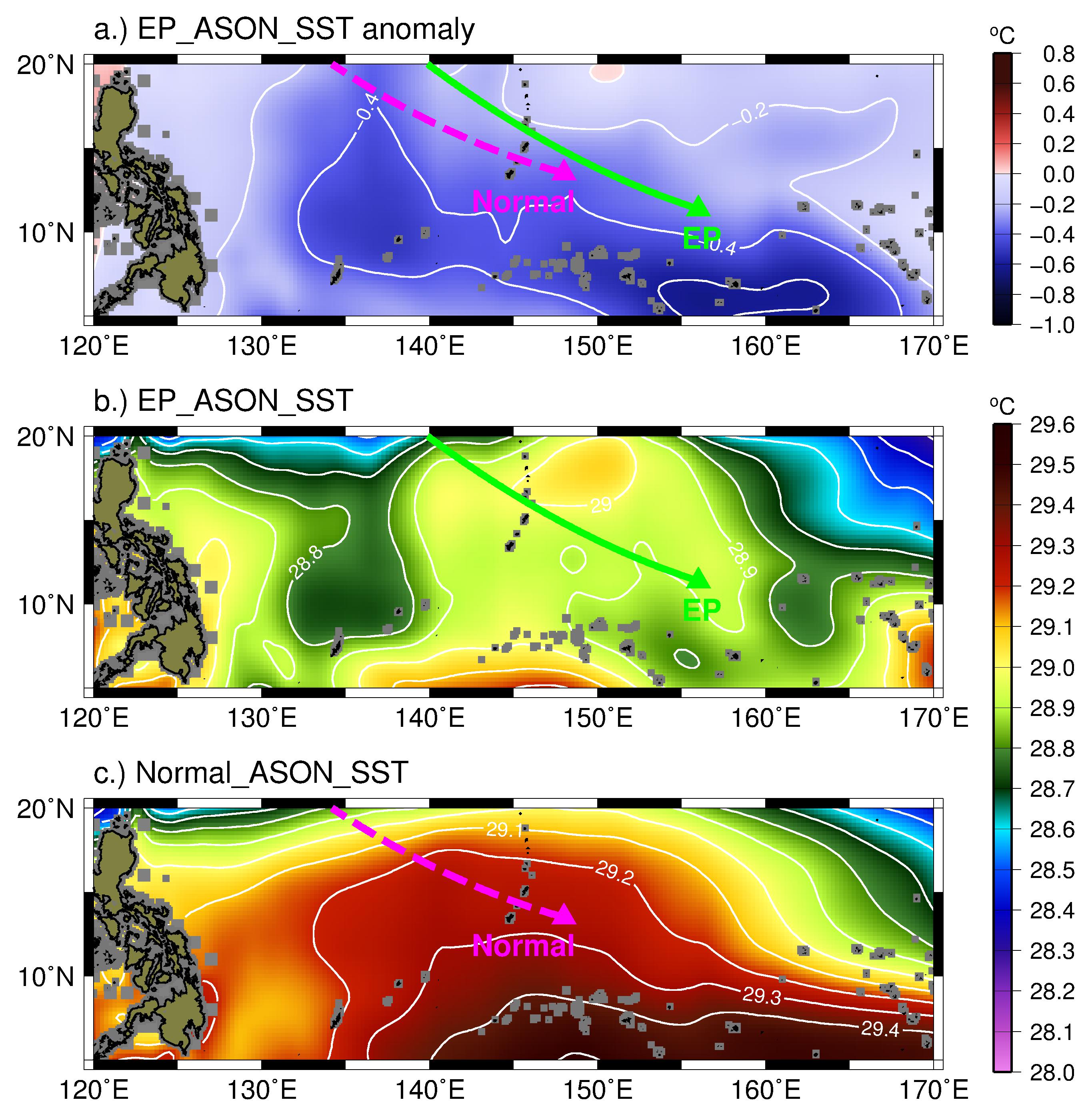
Fig. S11. As in Fig. S3, but for the SST of the 3 EP El Niño composites.


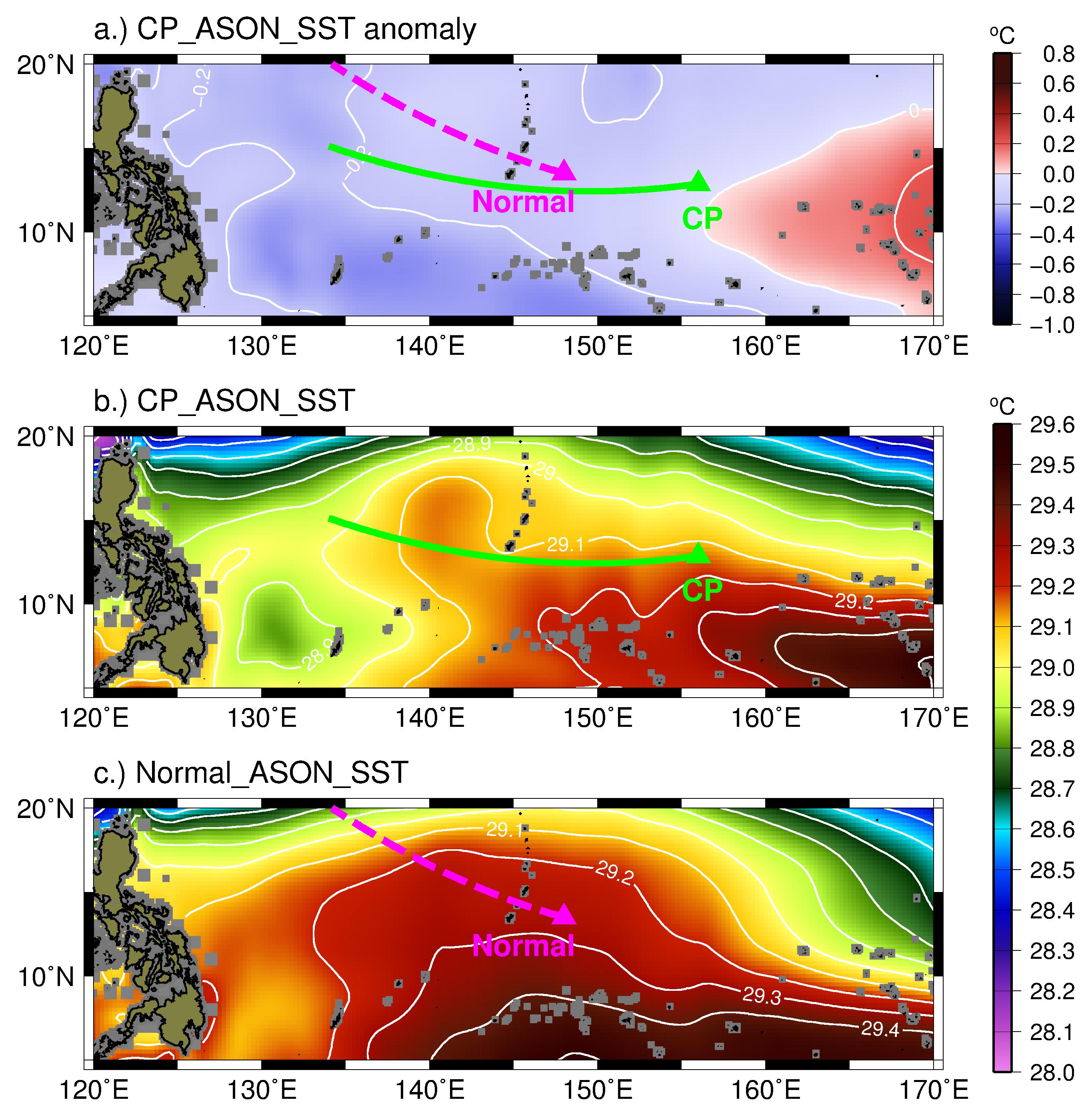
Fig. S12. As in Fig. S4, but for the SST of the 5 CP El Niño composites.


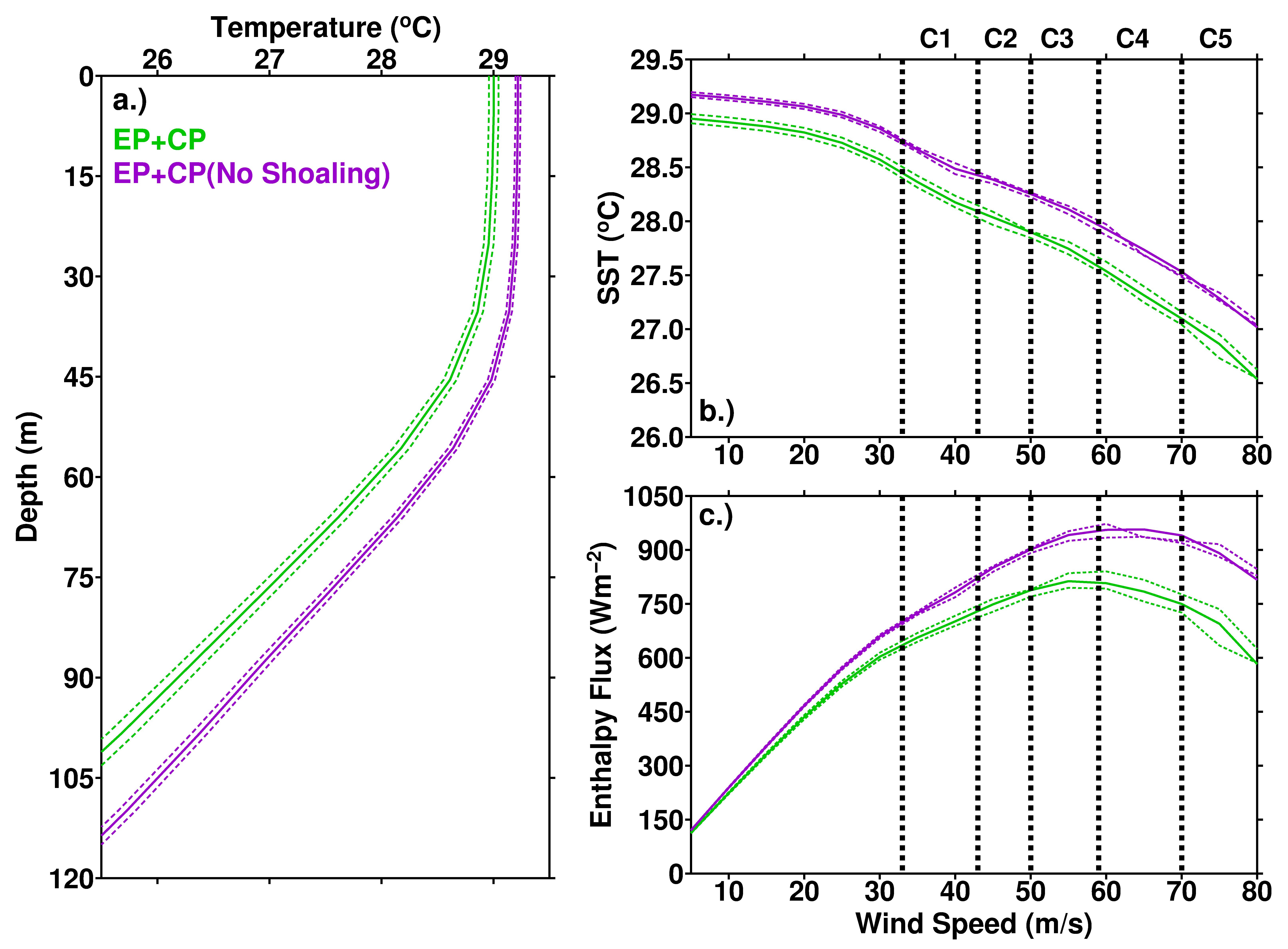
 Fig. S13. As in the Fig. 2 of the main text, but for the results from the 8 El Niño composites. **(a)** The initial, pre-TC ocean depth-temperature profile (green profile) averaged along the TC mean intensification track (location see in Figs. 1c and S2). For comparison, the purple profile is the initial profile under the ‘if no-shoaling’ scenario (based on the 1980-2010 ASON average), obtained along the same track. The dashed-profiles are the corresponding 95% confidence interval profiles from the mean. **(b)** During-intensification (from category-1 to 5 in the Saffir-Simpson scale) SST cooling as estimated using the Price-Weller-Pinkel 3-D ocean mixed layer model. The initial input was based on the 2 profiles in (a). **(c)** As in (b), but for the corresponding air-sea enthalpy (latent + sensible) flux supply.


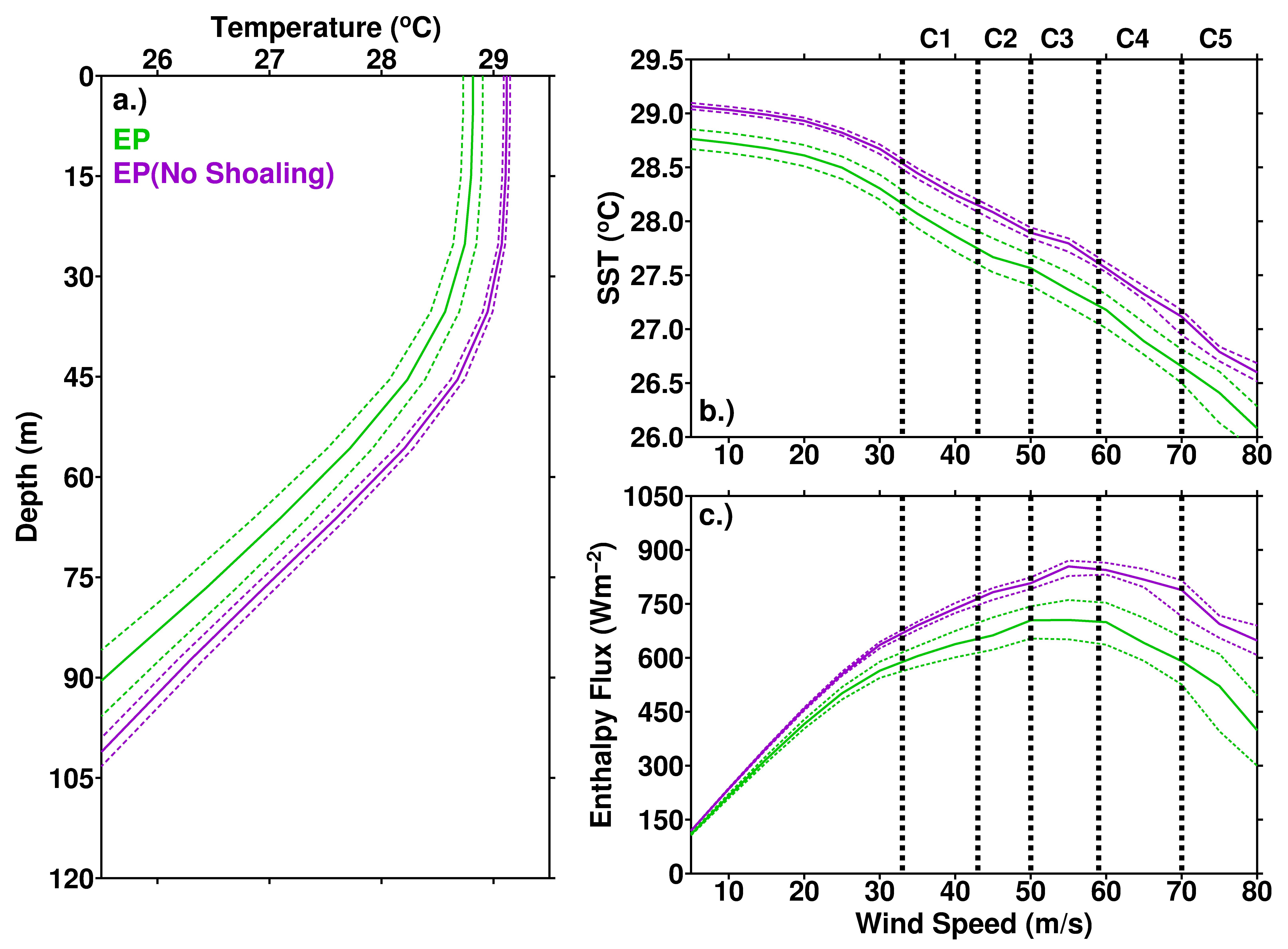
Fig. S14. As in Fig. S13, but for the results from the 3 EP El Niño composites.


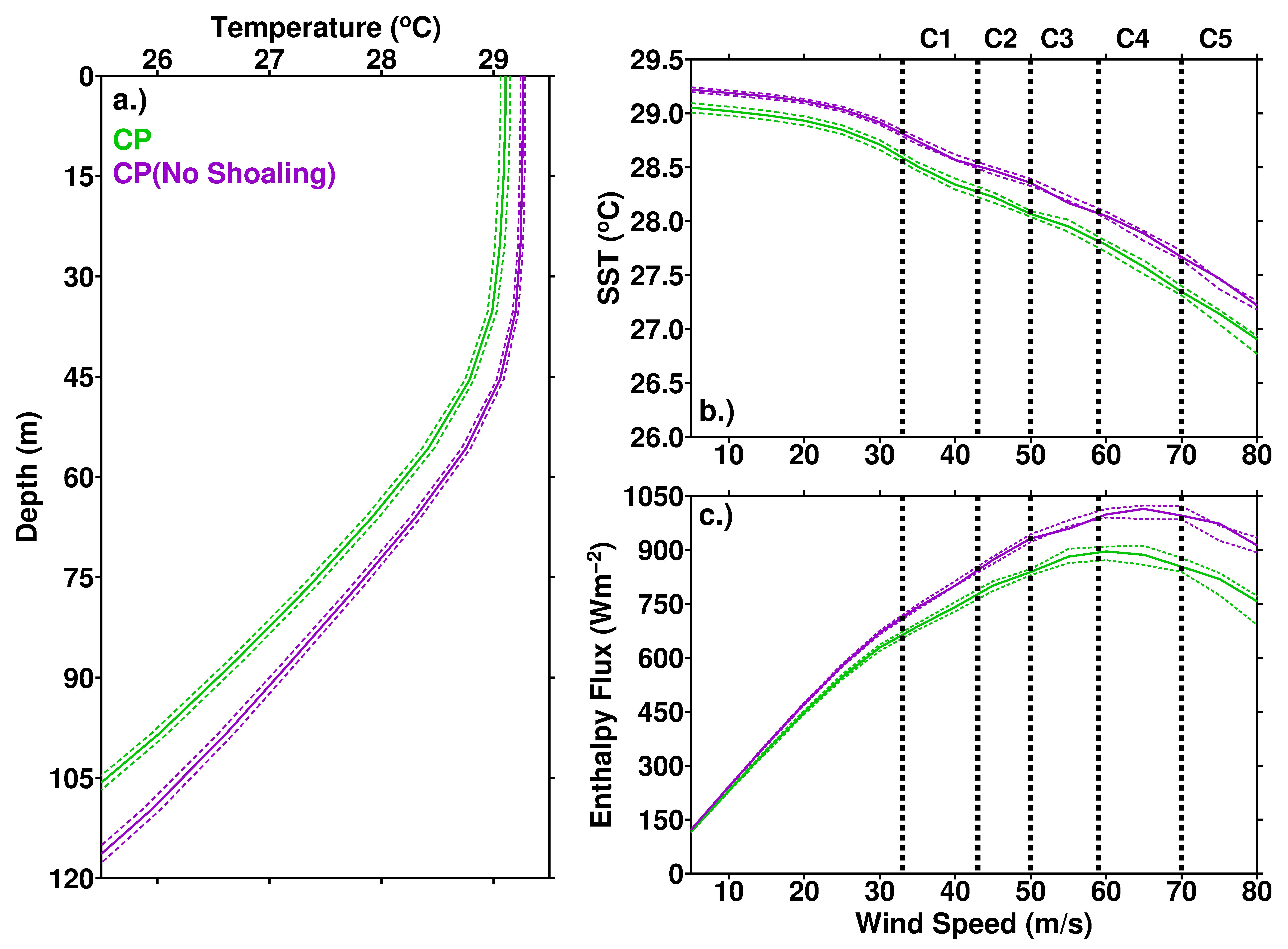
Fig. S15. As in Fig. S13, but for the results from the 5 CP El Niño composites.


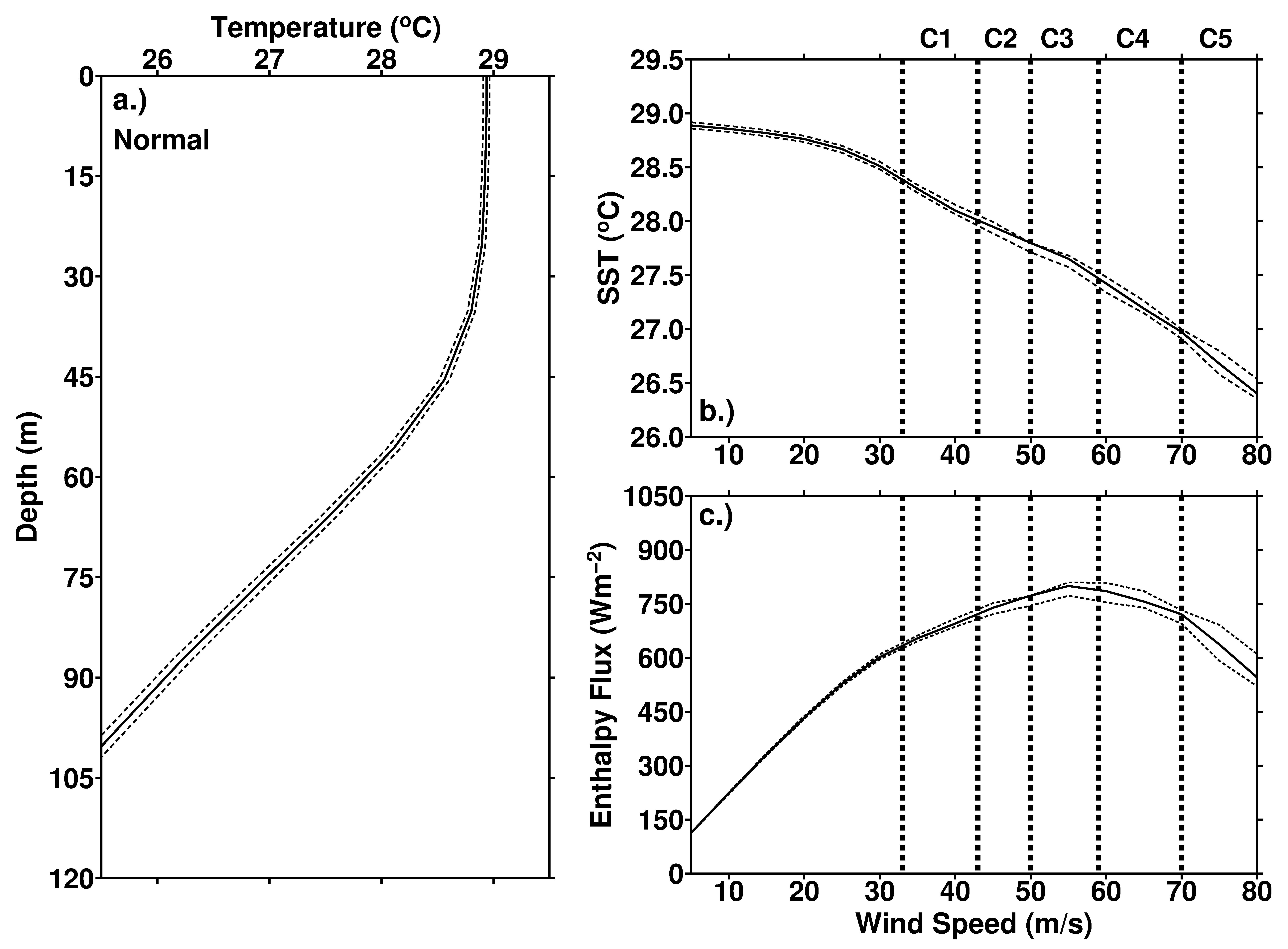
 Fig. S16. For reference, the normal ocean pre-TC initial condition (during ASON, 1980-2010 average) along the climatology intensification track (pink mean track in Fig. 1c). The dashed-profiles are the corresponding 95% confidence interval profiles from the mean. **(b)** During-intensification (from category-1 to 5 in the Saffir-Simpson scale) SST cooling as estimated using the Price-Weller-Pinkel 3-D ocean mixed layer model. The initial input was based on (a). **(c)** As in (b), but for the corresponding air-sea enthalpy (latent + sensible) flux supply.


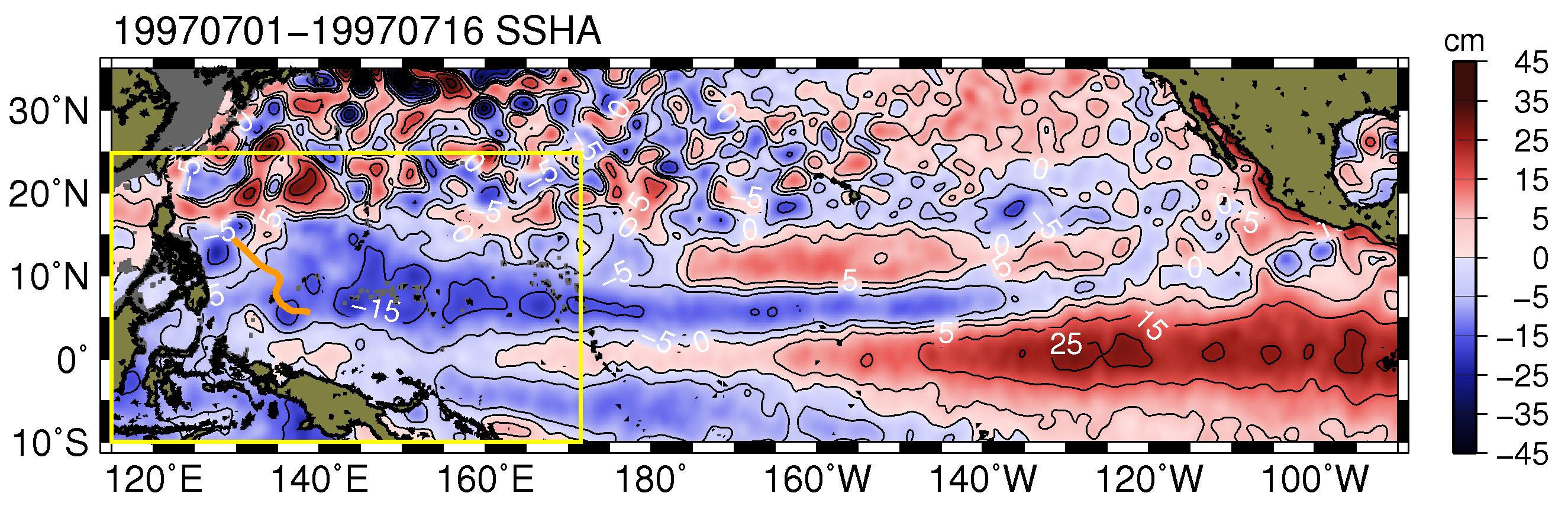
Fig. S17. Satellite SSHA map observed in early July 1997, showing the pre-existing SSHA condition prior to the 1^st^ super-typhoon Rosie (intensification location depicted as orange track).

**
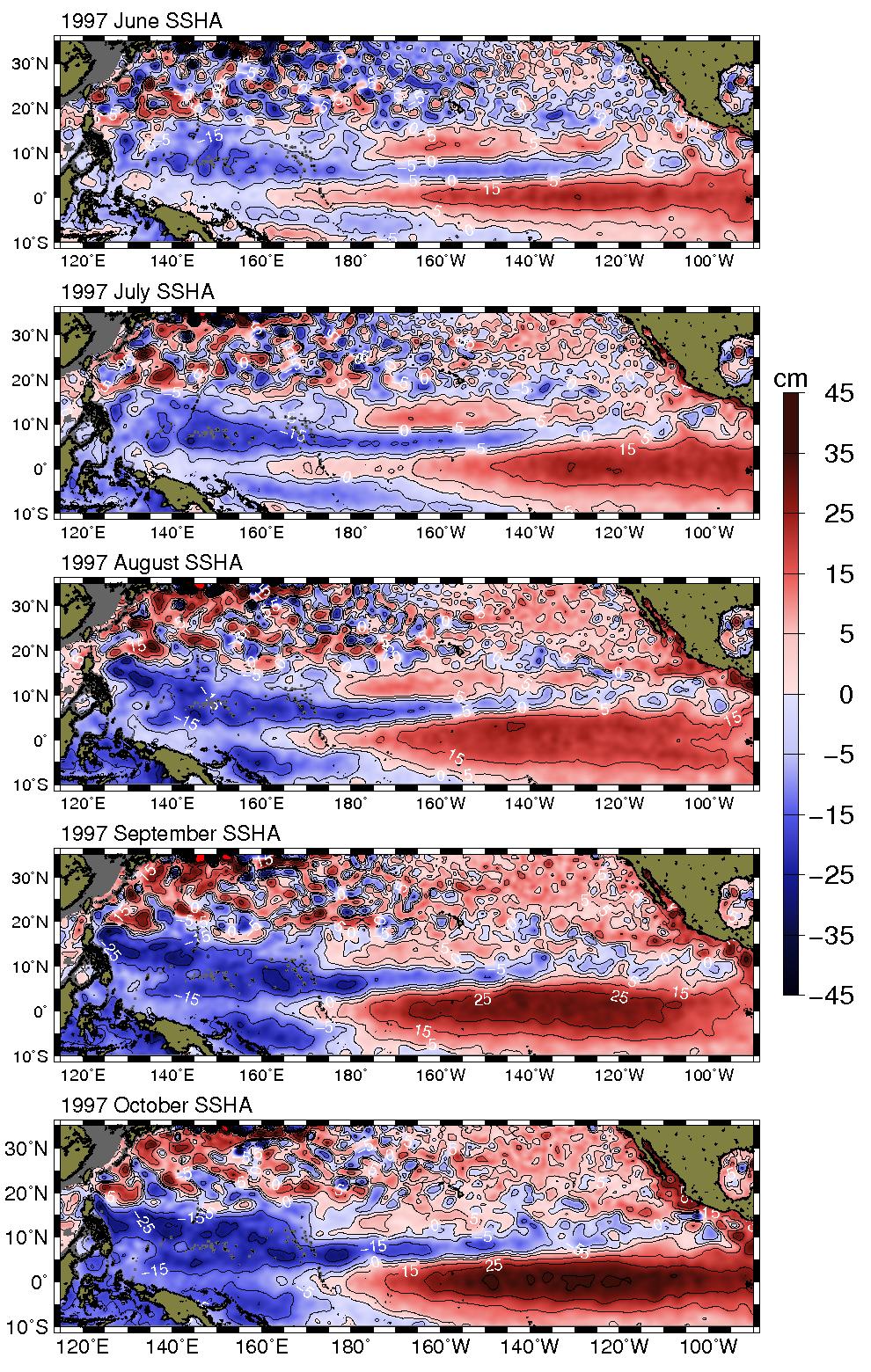
**

**Fig. S18.** Satellite SSHA map of the tropical Pacific Ocean from June to October 1997, showing the development of the shoaling effect over the WNPO, with negative SSHA strengthening.

**Table_S1:** Statistical results of the 3 ocean parameters (SST, D26, UOHC) under the climatological (1980-2010 average) condition.

| **Control : Climatology** | **SST (˚C)** | **D26 (m)** | **TCHP**  **(KJ/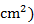** |
| --- | --- | --- | --- |
| **Mean** | 29.15 | 93 | 95 |
| **Standard Deviation** | 0.38 | 22 | 25 |
| **Standard Error** | 0.0025 | 0.1434 | 0.1661 |
| **95% confidential interval** | 0.005 | 0.289 | 0.326 |

**Table_S2:** As in Table_S1, but under the 1997 El Niño condition. Confidence level (in %) testing the statistical significance between El Niño and climatology (control) is also shown.

| **Case : 1997** | **SST (**˚C) | **D26 (**m) | **TCHP** (KJ/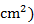 |
| --- | --- | --- | --- |
| **Mean** | 28.96 | 68 | 64 |
| **Standard Deviation** | 0.23 | 19 | 17 |
| **Standard Error** | 0.0079 | 0.7076 | 0.6248 |
| **95% confidential interval** | 0.016 | 1.387 | 1.225 |
| **T-test value (compared with control)** | 3.56 | 5.92 | 7.88 |
| **Confidence level (%)** | > 99.96 % | > 99.99 % | > 99.99 % |

**Table_S3:** As in Table_S2, but under the 8 El Niño composite condition.

| **Case : EP2+CP2** | **SST** | **D26** | **TCHP** |
| --- | --- | --- | --- |
| **Mean** | 28.96 (˚C) | 80 (m) | 76 (KJ/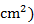 |
| **Standard Deviation** | 0.31 | 20 | 18 |
| **Standard Error** | 0.0040 | 0.2531 | 0.2290 |
| **95% confidential interval** | 0.008 | 0.496 | 0.449 |
| **T-test value (compared with control)** | 4.09 | 4.36 | 6.71 |
| **Confidence level (%)** | > 99.99 % | > 99.99 % | > 99.99 % |

**Table_S4:** As in Table_S2, but under the 3 EP El Niño composite condition.

| **Case : EP2** | **SST** (˚C) | **D26** (m) | **TCHP** (KJ/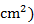 |
| --- | --- | --- | --- |
| **Mean** | 28.87 | 76 | 69 |
| **Standard Deviation** | 0.30 | 21 | 19 |
| **Standard Error** | 0.0053 | 0.4434 | 0.3924 |
| **95% confidential interval** | 0.012 | 0.869 | 0.769 |
| **T-test value (compared with control)** | 5.35 | 4.66 | 7.69 |
| **Confidence level (%)** | > 99.99 % | > 99.99 % | > 99.99 % |

**Table_S5:** As in Table_S2, but under the 5 CP El Niño composite condition.

| **Case : CP2** | **SST** | **D26** | **TCHP** |
| --- | --- | --- | --- |
| **Mean** | 29.01 (˚C) | 83 (m) | 80 (KJ/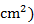 |
| **Standard Deviation** | 0.31 | 18 | 16 |
| **Standard Error** | 0.0050 | 0.2991 | 0.2592 |
| **95% confidential interval** | 0.010 | 0.586 | 0.508 |
| **T-test value (compared with control)** | 2.741 | 3.417 | 5.281 |
| **Confidence level (%)** | > 99.4 % | > 99.94 % | 99.99 % |
